# Foundation and methodologies in computer-aided diagnosis systems for breast cancer detection

**DOI:** 10.17179/excli2016-701

**Published:** 2017-02-20

**Authors:** Afsaneh Jalalian, Syamsiah Mashohor, Rozi Mahmud, Babak Karasfi, M. Iqbal B. Saripan, Abdul Rahman B. Ramli

**Affiliations:** 1 Department of Computer and Communication Systems Engineering, Faculty of Engineering, Universiti Putra, Malaysia; 2 Department of Imaging, Faculty of Medicine and Health Science, Universiti Putra, Malaysia; 3 Department of Computer Engineering, Qazvin Branch, Islamic Azad University, Qazvin, Iran

**Keywords:** breast cancer, computer-aided diagnosis system, segmentation, feature extraction, classification

## Abstract

Breast cancer is the most prevalent cancer that affects women all over the world. Early detection and treatment of breast cancer could decline the mortality rate. Some issues such as technical reasons, which related to imaging quality and human error, increase misdiagnosis of breast cancer by radiologists. Computer-aided detection systems (CADs) are developed to overcome these restrictions and have been studied in many imaging modalities for breast cancer detection in recent years. The CAD systems improve radiologists' performance in finding and discriminating between the normal and abnormal tissues. These procedures are performed only as a double reader but the absolute decisions are still made by the radiologist. In this study, the recent CAD systems for breast cancer detection on different modalities such as mammography, ultrasound, MRI, and biopsy histopathological images are introduced. The foundation of CAD systems generally consist of four stages: Pre-processing, Segmentation, Feature extraction, and Classification. The approaches which applied to design different stages of CAD system are summarised. Advantages and disadvantages of different segmentation, feature extraction and classification techniques are listed. In addition, the impact of imbalanced datasets in classification outcomes and appropriate methods to solve these issues are discussed. As well as, performance evaluation metrics for various stages of breast cancer detection CAD systems are reviewed.

## Introduction

Breast cancer is the most common cause of death after lung cancer in the world. Early detection and efficient treatment of breast cancer could increase the treatment options and decline the mortality rate. Different modalities such as mammography, ultrasound, and Magnetic Resonance Imaging (MRI) are the most effective tools in the early detection of breast cancer. Issues such as technical reasons, which are related to imaging quality and human error have increased the misdiagnosis of breast cancer in radiologists' interpretation. In the effort to overcome such restrictions, CAD systems are developed to automated breast cancer detections and classify benign and malignant lesions. The CAD systems improve radiologists' performance in finding and discriminating between the normal and abnormal tissues. These procedures are performed only as a double reader but the absolute decisions are still made by the radiologist.

Recent advances in the resolution of medical imaging modality have revolutionised the diagnostic accuracy. Efficient usage of imaging data to improve the diagnosis is very important. In recent years, computer-aided diagnosis systems (CADs) have developed a new context in radiology to take advantage of the data to be applied to different imaging modality and the diagnosis of different diseases. The efficiency of radiologists' interpretation can be improved in terms of accuracy and consistency in detection or diagnosis while productivity can be improved by reducing the time required to read the images (Doi, 2009[36]). The outcomes are derived using various techniques in computer vision to present some of the significant parameters such as the location of suspicious lesions and the likelihood of malignancy of the detected lesions.

In relation to breast cancer, the main objective of CAD system is to design accurate and reliable approach, to decrease observational oversights and assist in discriminating benign and malignant lesions. In the following, we present some of the recent proposed CAD systems for breast cancer detection or diagnosis on various modalities such as mammography, ultrasound, MRI, and biopsy histopathological images. 

### Mammography

Mammography is a dedicated imaging modality for breast screening that uses low-dose X-ray during breast examination. Mammography is currently the most effective tool for early detection of breast cancer; however, it has some restrictions. Breast density is a variety of confounding factors that make diagnosis of breast cancer more difficult in women with dense breasts (Ertosun and Rubin, 2015[42]). The contrast between cancer and background in dense breast image is very low, which can affect the diagnosis outcome (Longo et al., 2014[89]). In the mammographic examination, non-cancerous lesions can be misinterpreted as cancer (false-positive value), while cancers may be missed (false-negative value). As a result, radiologists fail to detect 10 % to 30 % of breast cancers (Bird et al., 1992[14]; Boyd et al., 2007[16]; Kerlikowske et al., 2000[74]). The false-positive value indicates the percentage of lesions that are found to be cancerous and subjected to biopsy. The miss rate in mammography has increased in dense breasts where the probability of cancer is four to six times higher than in non-dense breasts (Boyd et al., 2007[16]; Maskarinec et al., 2007[94]; Nelson et al., 2009[102]). Several solutions have been proposed to enhance the specificity and sensitivity of mammography as well as to decrease unnecessary biopsies procedure.

Double reading is one of the solutions that can significantly contribute to achieving high sensitivity and specificity (Dinnes et al., 2001[35]; Warren and Duffy, 1995[135]). Additional costs will be imposed on the patients for double reading of mammography. CAD systems can be considered as an alternative framework that acts as a second reader to enhance the performance of physician's interpretation. The studies (Balleyguier et al., 2005[11]; Sanchez Gómez et al., 2011[117]; Malich et al., 2000[91]; Marx et al., 2004[93]) have shown that the attention to use a computer to improve the performance of physicians to detect mass and micro-calcification in mammography has increased in recent years. Gilbert et al. (2008[49]) indicated that proportion of cancer detected was 199 of 227 (87.7 %) for double reading and 198 of 227 (87.2 %) for single reading with CAD system. The perspective assessment of the impact of CAD systems on interpretation mammogram images has been performed on a community of breast cancer patients (Freer and Ulissey, 2001[46]). Among 12,860 mammograms, the radiologist's performance was measured without CAD and with CAD. The recall rate increased from 6.6 % to 7.7 % and the proportion of early-stage malignancy detected the growth from 73 % to 78 %, which represents an increase in efficiency in the detection of cancer with the usage of CAD system.

Micro-calcifications and masses are the two most significant signs of malignancy. Breast calcification is tiny specks of calcium which are scattered in the breast. In order to classify micro-calcification in benign and malignant, different properties such as size, shape, distribution pattern, density, and a number of micro-calcifications are analysed (Mousa et al., 2005[100]). Detection of micro-calcifications is a difficult task and the hardship increases in mammogram interpretation in young women due to the contrast reduction among micro-calcification and adjacent tissue (Nunes et al., 2007[105]). Rizzi et al. (2012[112]) have provided a valuable study on techniques for suppressing noise, enhancing contrast, and extraction; and classification of micro-calcification. 

Another lesion in the breast is mass, which; is a circumscribed lump in the breast and might be categorised to be benign or malignant. Masses are characterised by various attributes such as shape (round, lobular, oval, irregular), margin (obscured, indistinct, and speculated), size, location, and their contrast. Mass detection is more difficult compared to micro-calcification because of the similarity and ambiguity of their characteristics with the normal tissue (Islam et al., 2010[66]; Kozegar et al., 2013[77]). Masses are generally observed in the dense regions of the breast with smoother boundary rather than micro-calcification (Kozegar et al., 2013[77]). Due to these factors, mass detection is a challenging task for radiologists. In the past two decades, researchers have conducted a lot of effort for developing automatic systems to help radiologists in the detection and diagnosis of mass on mammography image. Oliver et al. (2010[107]) have presented an exhaustive study of CAD systems for the detection and segmentation of mass from mammogram images. In this study, the introduction of current systems for mass detection and the used strategies as well as a quantitative comparison of a few methods are provided.

Although mammography is a proven modality for mortality reduction in breast cancer, one of the noteworthy points is low sensitivity and specificity in young women and dense breast (Boyd et al., 2007[16]; Maskarinec et al., 2007[94]; Nelson et al., 2009[102]). Low specificity in screening mammography may cause some unnecessary biopsy (Jesneck et al., 2007[71]). This restriction increases the cost and stress imposed on the patient. Consequently, to gain high precision in mammography screening alone is difficult. Some observational studies have shown improve screening sensitivity in women with dense breast, through adjunct mammography with ultrasound (Berg et al., 2008[12]; Corsetti et al., 2011[28]; Drukker et al., 2013[40]; Nothacker et al., 2009[104]; Ohuchi et al., 2016[106]; Scheel et al., 2015[119]).

### Ultrasound

Ultrasound is a beneficial tool to evaluate breast issues and to follow up finding in physical exam or mammography. It is also recommended for breast screening during pregnancy and lactation. Ultrasound is suggested before diagnostic fine needle biopsy and it can be used for biopsy guidance and mass locating. Although ultrasound is less sensitive than MRI, it has converted a valuable tool as an adjunct to mammograms due to its availability, non-invasive, and costs effective than other options. The development of colour Doppler imaging and ultrasound echo-enhancing (contrast agents) provides additional information of anatomical and vascular flow related, which assists the differential diagnosis of breast lesions (Svensson, 1997[128]).

The studies indicate that ultrasound is able to detect and discriminate benign and malignant masses with high accuracy and also reduce the number of unnecessary biopsies (Chen et al., 2003[25]; Sahiner et al., 2007[116]). Ultrasound is more sensitive for detecting invasive cancer in dense breasts (Costantini et al., 2006[29]; Drukker et al., 2002[39]). However, it is an operator-dependent modality and the interpretation of its images requires expertise on the part of the radiologist. In order to overcome the operator dependency and increase the accuracy of diagnosis rate, computer-aided detection/diagnosis (CAD) systems are developed for breast cancer detection and classification on ultrasound images. Recently, several CAD systems have been proposed to reduce the influence of dependence on the operator in ultrasound and increase the diagnosis sensitivity and specificity (Huang et al., 2004[64], 2006[65]; Kim et al., 2014[75]). CAD systems have been presented on Automated Breast Ultrasound (ABUS) (Kim et al., 2014[75]). The CAD system is evaluated on a dataset that involves 20 cysts, 42 benign lesions, and 27 malignant lesions. The sensitivity achieved by this system was 82.67 percent and the false positive rate was 0.26 per image.

The efficiency of CAD software is generally higher to detect lesion with a high risk of malignancy in contrast to the benign lesion (Chabi et al., 2012[20]; Horsch et al., 2004[62]). Chabi et al. (2012[20]) have presented the results of ultrasound image analysis with and without CAD system by junior radiologists to detect breast cancer. With the high sensitivity (95 %) and low specificity (48 %) achieved in this research, it appears that CAD system is a useful tools for image interpretation for the junior radiologist in training. 

### Magnetic Resonance Imaging (MRI)

Since nearly three decades, MRI screening has been employed for detection and diagnosis of breast cancer lesions (Heywang et al., 1989[60]). Breast MRI is a potential alternative, but the cost is higher than other imaging methods and not widely available as ultrasound and mammography. MRI is suggested for screening women who have a high risk of developing breast cancer, or it can be used to investigate suspicious areas found by the mammogram to help measure the size of the mass. Breast MRI is advised to women with family history of breast cancer and has a high rate of sensitivity (78-98 %) and low specificity ( 43-75 %) (Kuhl, 2007[80]). The interpretation process of MRI image is very time-consuming and requires a high level of radiologist experience to detect and differentiate benign and malignant lesions (Meeuwis et al., 2010[95]). In recent studies, computer systems have been developed to facilitate MRI image analysis and improve the diagnosis productivity (Meeuwis et al., 2010[95]; Wang et al., 2009[134]; Williams et al., 2007[136]). 

### Biopsy

Biopsy is the final stage when a mammogram or other imaging modalities found any type of abnormality. During a biopsy, a sample is taken from suspicious lesion in order to conduct microscopic observation. There are several types of biopsies such as fine needle aspiration biopsy (FNAB), core biopsy, or surgical biopsy. FNAB is a common type of biopsy and during the examination; a cytological sample is obtained from the tumour and explored under a microscope to determine the occurrence of cancer cells. The main disadvantage of FNAB is that the needle cannot extract sufficient amount of tissue for diagnosis. Detection of cancer cells requires profound knowledge and sufficient experience in the field of histopathology (Filipczuk et al., 2012[45]). A vision-based computer system to automatically detect the cancer cells can help specialists to discriminate cancer from non-cancer cells.

In contrast to other CAD systems, fewer studies performed the analysis of breast histopathology images. Issac Niwas et al. (2012[67]) have used Log-Gabor wavelet transform base decomposition method for histopathological images on HSV (Hue, Saturation, Value) colour space. The accuracy obtained by Least squares Support Vector Machine (LS-SVM) in this study was 98.3 %. Another study has applied the Genetically Optimized Neural Network (GONN) algorithm for diagnosis on histopathology images (Bhardwaj and Tiwari, 2015[13]). They achieved an average accuracy of 97.73 %, 99.11 %, and 99.21 % for 50-50, 60-40 and 70-30 training-testing partitions respectively, and 99.26 % for 10-fold cross validation structure.

## Cornerstones of a CAD System

**Figure 1:**
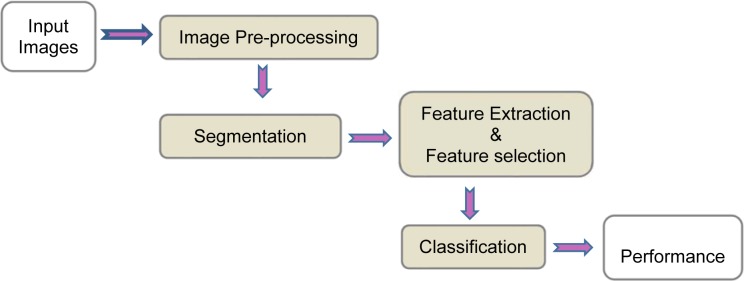
Overall view of CAD system for breast cancer diagnosis

Medical image processing requires prior knowledge on the content and nature of image to select appropriate methods to implement the CAD system. In order to achieve a high level of efficiency for automated diagnosis, it is significant to employ efficacious image processing approaches in the main steps of CAD system. Commonly, the CAD systems consists of four stages as shown in Figure 1(Fig. 1).

A brief description of the main stages of a CAD system is provided as follows:

1. Image pre-processing: This step is essential for some modality such as ultrasound for the purpose of enhancing the image and reducing the noise with minimum distortion of image features. Some of the CAD systems do not have a pre-processing stage.
2. Image segmentation: Image segmentation is a vital step towards efficient development of CAD systems. The main purpose of segmentation is the separation of the region of interest (ROI) commensurate with the desired properties (Nie, 2009[103]). Recently, imaging modalities such as magnetic resonance imaging (MRI), computed tomography (CT), 3D ultrasound, and many more modalities are capable of producing images in the form of 3D. Therefore, 3D segmentation methods are desirable for more accurate segmentation in volumetric imagery.
3. Feature extraction and selection: In this step, different features are extracted according to the characteristics of lesions from the image. These features are used to distinguish benign or malignant lesions. The feature set is usually very large and the selection of the most effective features is very critical for the next step.
4. Classification: According to the selected features, the suspicious areas are classified to benign or malignant based on different classification techniques. The common classification methods used in medical imaging are presented in this section.
5. Performance evaluation: This step evaluates the performance of CAD system.

### Image pre-processing

In medical image processing, image pre-processing plays a significant role to achieve the ideal outcomes in other stages of a CAD system such as segmentation and feature extraction. Pre-processing stage is performed to remove noise and defect caused in image acquisition procedure, image resizing, and enhance the image intensity (Kyaw, 2013[82]).

### Image segmentation

Image segmentation is a crucial component in computer vision and pattern recognition. Accurate segmentation plays a significant role in the success or failure of the computerised procedure. In medical imaging aspect, the selection of segmentation methods are widely depending on the specific application and imaging modality. With increased dimension and resolution of the image in various modalities, the images cannot be manually examined with regard to the huge amount of image information. Segmentation techniques help to highlight significant regions and extract various structures such as organs or tumours for further examination.

Segmentation methods are categorised generally into two groups: semi-automatic and fully automatic. Providing an automatic algorithm in medicine to detect and localise abnormality is highly desirable. In medical application, the low rate of false positive and false negative detection is very important. Therefore, evaluation methods of segmentation algorithms is another dimension of CAD systems in the practical aspect. 

**Figure 2:**
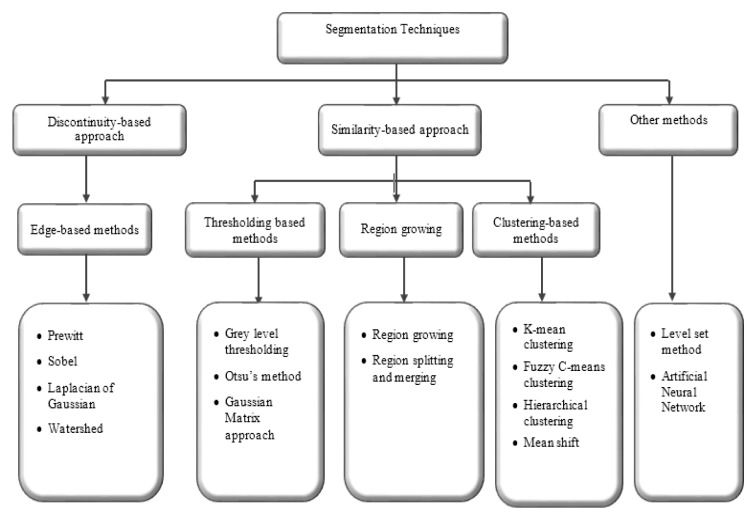
Overall view of segmentation techniques

Segmentation approaches based on image properties are broadly arranged into two groups: discontinuity-based approach and similarity-based approach. Discontinuity-based approach partitions an image based on an abrupt change in intensity (Rastgarpour and Shanbehzadeh, 2011[111]), while similarity-based method partitions an image according to pre-determined similarity criteria. The similarity-based method is categorised into region-based, thresholding-based, and clustering-based methods (Lee et al., 2015[83]). The overall view of segmentation techniques is shown in Figure 2(Fig. 2). A general comparison of segmentation methods (Lee et al., 2015[83]; Narkhede, 2013[101]) are provided in Table 1(Tab. 1). 

#### Edge-based segmentation methods

Edge-based segmentation methods are a structural technique to detect edges or pixels among different regions that have abrupt intensity change (Pal and Pal, 1993[108]). The edge-based method works well on high contrast and non-noise images. There are several methods for edge-based segmentation such as Sobel, Prewitt, Laplace, Canny, and Laplacian of Gaussian (Dromain et al., 2013[38]). The main application of edge-based segmentation techniques is human organ recognition. A mathematical morphological edge detection algorithm has been presented in (Yu-qian et al., 2006[139]) to detect lungs in CT images that contain salt-and-pepper noise. Haris et al. (1998[56]) have proposed an integration of edge-based and region-growing with watershed transform for 2D/3D segmentation of magnetic resonance images.

#### Thresholding-based segmentation methods

One of the wide methods used for image segmentation is a thresholding-based technique which is an effective way to discriminate foreground from the background image (Zhang, 2006[141]). The first step in this method is the selection of an appropriate threshold value according to image properties, and then the pixels image is assigned to specific regions. The automatic selection of threshold value requires the knowledge on the intensity characteristics of the objects, sizes of the objects, and the number of various types of objects existing in the image (Al-Amri and Kalyankar, 2010[4]). 

The thresholding-based methods have been widely used to develop CAD systems in order to extract significant areas for additional analysis. In the article of Al-Bayati and El-Zaart (2013[5]), various thresholding techniques have been compared to segmentation mammogram images. An automatic nucleus segmentation is developed on the image of breast histopathology using histogram-based thresholding (Saha et al., 2015[115]). The result shows 97 % accuracy in nucleus detection.

#### Region growing segmentation methods

The region-based segmentation methods divide an image into homogeneous regions of connected pixels based on predefined criteria such as intensity, colour, or texture. Region-based approaches are broadly classified into two groups: region growing and region split and merging (Narkhede, 2013[101]). Seed selection is the first step in the region growing segmentation methods. The initialisation of seed-point is crucial and effective on the result of segmentation. In contrast with the region growing, region split and merging is a top-down approach. The procedure starts with a whole image and partitioning to achieve more homogeneous regions. Appropriate regions with similar criterions can be merged into one region.

In recent years, region-based segmentation algorithms are widely used to develop CAD systems for breast cancer detection. A region growing approach is applied to the extraction of a region of interests (ROIs) in this article (Rouhi et al., 2015[113]). Another adaptive region growing scheme with the composition of hybrid assessment function, maximum likelihood analysis, and maximum gradient analysis is produced in this study (Cao et al., 2010[18]). Split and merging algorithm is employed in this paper (Rundo et al., 2016[114]) based on the seed selection by an adaptive region growing procedure.

#### Clustering-based segmentation methods

Clustering-based segmentation methods are popular techniques in medical image segmentation and generally categorised into hierarchal and partitional clustering (Jain, 2010[68]). Hierarchal clustering algorithms are a recursive process which is able to find nested clusters in agglomerative (bottom-up) or divisive (top-down) method. In comparison with hierarchal clustering, partitioning clustering techniques are an iterative procedure which can be divided into hard clustering and fuzzy clustering (De Carvalho et al., 2012[30]). In hard partitioning methods, each object is allocated in only one cluster, whereas, in fuzzy clustering, a set of membership levels associated with each element and each element can belong to more than one cluster. K-means and fuzzy C-means clustering are well-known techniques for hard and fuzzy clustering respectively. 

Clustering methods are potentially very beneficial in clinical image segmentation. Authors have presented adaptive K-means clustering technique (Moftah et al., 2014[97]) and Fuzzy C-Means (FCM) clustering (Sathya et al., 2012[118]) on MRI breast images. In Table 2(Tab. 2) (References in Table 2: Al-Faris et al., 2014[6]; Kim et al., 2014[75]; Dheeba et al., 2014[34]; Al-Faris et al., 2014[7]; Hassanien et al., 2014[57]; Kannan et al., 2011[73]) a few examples of segmentation methods in breast CAD systems collected are shown. 

Recently, imaging modalities such as magnetic resonance imaging (MRI), computed tomography (CT), 3D ultrasound, CTLM and many more modalities are capable of producing images in the form of 3D. Therefore, 3D segmentation methods are desirable for more accurate segmentation in volumetric imagery. Some approaches have been proposed on different modalities such as breast MRI (Song et al., 2015[124]) and automated 3D breast ultrasound (Moon et al., 2013[98]; Tan et al., 2015[129], 2013[130]).

The development of reconstruction algorithms has been the attention of many researchers in latest work since the reconstruction of 3D images for consecutive images, due to its various applications in surgery, teaching anatomy, and diagnosis (Li et al., 2015[85]). In the field of breast screening, segmentation and 3D reconstruction of Magnetic Resonance Image (MRI) (Gnonnou and Smaoui, 2014[50]) and mammogram (Yong et al., 2015[138]) are offered for breast cancer detection. 

### Feature extraction

**Figure 3:**
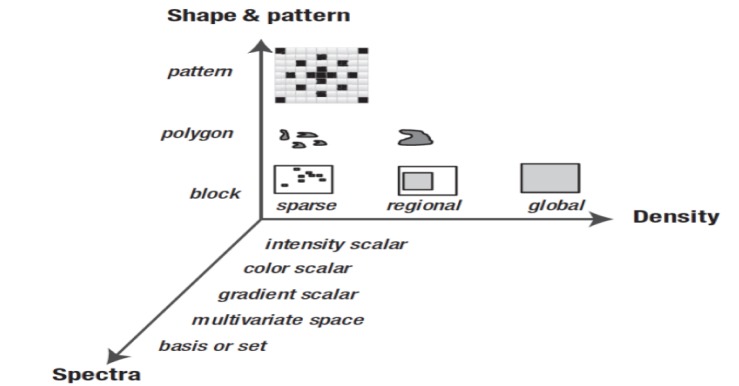
Taxonomy for feature descriptor dimensions (Krig, 2014)

Computing feature descriptors from an image to reduce the volume of data ordinarily signifies feature extraction. Features are characteristics of the whole image or region of interests. The proper selection of features has an important influence on (1) memory size, (2) accuracy of classification, (3) computational cost of classification and (4) robustness. Feature descriptors and metrics widely depend on the specific application. Generally, image descriptors are divided into the three dimensions. Figure 3(Fig. 3) (Reference in Figure 3: Krig, 2014[78]) shows a feature taxonomy (Krig, 2014[78]) based on feature descriptor dimensions using three axes: shape, pattern and spectra, and density.

**Figure 4:**
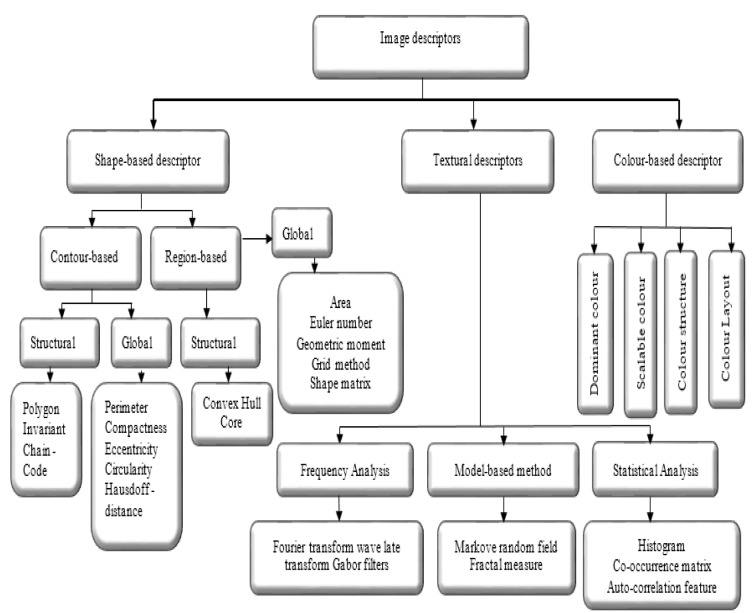
Overview of image descriptors

According to the literature (El Atlas et al., 2014[41]; Martinez, 2004[92]), image descriptors are placed in three categories, namely shape-based, textural, and colour-based descriptor, which is shown in Figure 4(Fig. 4). Shape features are the important properties employed by human to discriminate objects with other features such as colour and texture. To address the complexity conversion of shapes, an effective shape descriptor should be invariant into the rotation and scaling. 

Texture features are the most common features that are utilised to analyse and interpret images by considering the variation of intensity and quantifying different properties such as smoothness, coarseness, and regularity (Kurani et al., 2004[81]). A brief survey of extant techniques on textured feature extraction is as follows: grey level co-occurrence matrix (Haralick et al., 1973[55]), grey level run length method (Chu et al., 1990[27]), texture based on the fractal dimension (Chaudhuri and Sarkar, 1995[22]) and texture features based on windowed Fourier filters (Azencott et al., 1997[10]). Texture based features are extensively used in variety of applications, particularly in the medical image analysis (Huang et al., 2013[63]; Liu, 2013[87]; Moon et al., 2011[99]; Sundararaj and Balamurugan, 2014[127]).

Colour-based descriptors are the significant visual cue for image retrieval and object recognition (Liu and Yang, 2013[86]). The colour descriptors in the current version of MPEG-7 (Martinez, 2004[92]) are grouped in a number of histogram based descriptor including (1) dominant colour, (2) scalable colour, (3) colour structure, and (4) colour layout descriptors. With the release of various medical imaging modalities that involve colour information such as, Cervicography, Dermoscopy, fundus photography, and gastrointestinal endoscopy, colour descriptors are important in medical image analysis applications (Celebi and Schaefer, 2012[19]). Colour-based descriptors are rarely used in the work done in the field of breast cancer detection.

#### Shape-based descriptors

**Figure 5:**
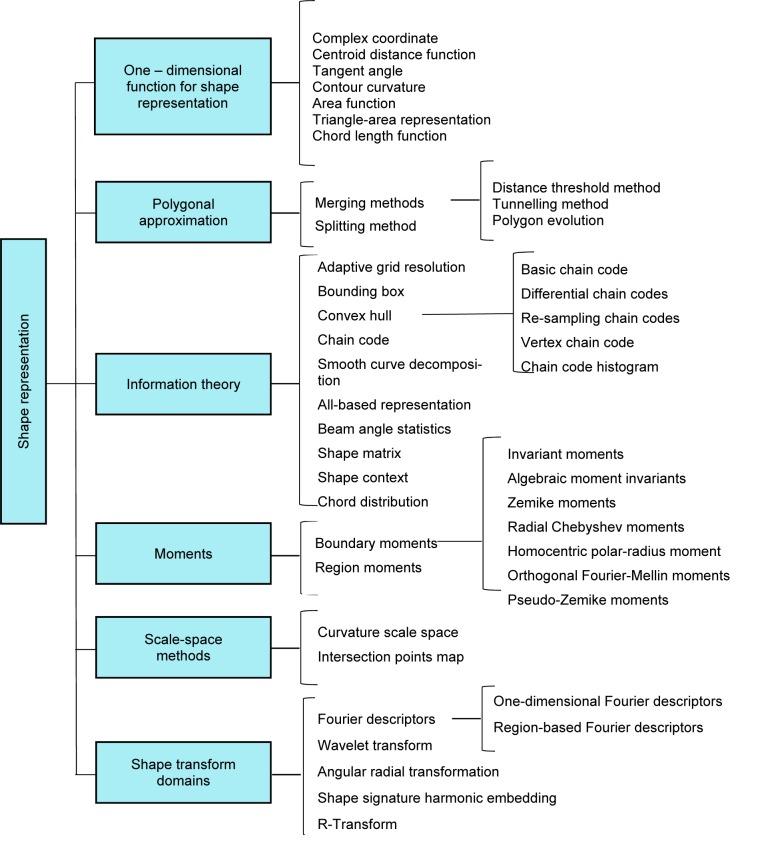
Overview of shape descriptor techniques (Yang et al., 2008)

Shape feature extraction plays a significant role in various application such as shape retrieval, shape recognition, and classification, and shape alignment and registration (Yang et al., 2008[137]). A hierarchical view of different shape description approaches collected is shown in Figure 5(Fig. 5) (Reference in Figure 5: Yang et al., 2008[137]).

The shape is one of the main visual cues in medical image processing, thus shape descriptors are widely used to design the feature extractors in CAD systems. In recent years, shape feature extraction has been receiving more attention in the case of breast cancer detection. In order to classify malignant or benign calcifications in mammograms, Shen et al. (1994[121]) have proposed a set of shape factors to measure the roughness of contours. Compactness, moments, and Fourier descriptor are extracted from the region of interests and the results show that higher accuracy is achieved by using a composition of the three shape factors. 

Zhang et al. (2012[140]), have developed a classifier to diagnose mass in mammograms. The mass shape features extracted from each contour contain area, convex, perimeter, circularity, compactness, solidity, convex, roughness, equivalent diameter, elongation, major axis length, minor axis length, eccentricity, and extent. The overall accuracy achieved in this study was 72 %.

To distinguish the exceptions in the shape of a lump in malignant and benign masses is a challenging task for physicians. A shape analysis method is presented by Rangayyan et al. (2000[110]) for the classification of masses in mammographic images. The extracted shape features in this study include concavity, convexity, speculation index and compactness. The obtained results in this work include the accuracy of 82 % and area (*A_z_*) of 0.79 under the receiver operating characteristics (James et al., 2001[69]) curve. 

In Gc et al. (2015[48]), three new shape features, namely convexity index, ircularity range ratio, and irregularity ratio are proposed to classify mammograms. The obtained accuracies in this work were 88 %, 87.5 %, and 94.5 % for convexity index, circularity range ratio and irregularity ratio, respectively. Radial distance measure (RDM), convexity and index angle are exploited from mass contour to feed into multilayer perceptron (MLP) and k-nearest neighbours (KNN) classifier (Boujelben et al., 2009[15]). The sensitivity obtained with MLP classifiers and KNN are 94.2 % and 93.7, respectively.

#### Textural descriptors

**Figure 6:**
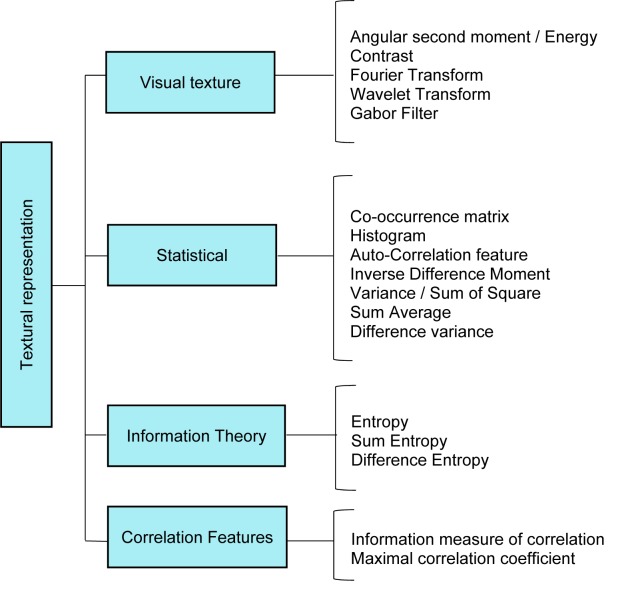
Overview of texture descriptor techniques

Texture features also are another expression of visual characteristics that is convenient for various domain of computer vision and pattern recognition. A hierarchical view of different texture description approaches is collected in Figure 6(Fig. 6).

Texture or pattern is another visual clue in medical image processing, which are widely applied to design the feature extractors in CAD systems. Recently, several researchers have used textural features for the development of breast CAD system. The co-occurrence texture feature is one of the significant features that are widely used in the design of CAD system. A fuzzy support vector machine is presented to classify mass using ultrasound images (Shi et al, 2010[122]).

Textural features consist of co-occurrence matrix based on spatial grey level dependence (SGLD), fractal features, and histogram-based features extracted and transferred into fuzzy support vector machine classifier (FSVM). The obtained accuracy, sensitivity and area (*A_z_*) in this work were 94.25 %, 91.67 % and 0.964, respectively. 

Filipczuk et al. (2012[45]) have employed the grey level co-occurrence matrix (GLCM) and grey level Run-length matrix (GLRLM) to develop an automatic breast cancer diagnosis system on cytological images of fine needle biopsy material. The results of classification show 90 % efficiency to detect malignancy in FNB breast image.

To conduct an analysis of the 3D morphology between benign and malignant tumours in breast MRI images, a set of texture features are used: GLCM features, Energy, Entropy, Correlation, Difference Moment, Inertia, Cluster Shade, Cluster Prominence, Horlick's Correlation (Huang et al., 2013[63]). The result shows the accuracy of 88.42 %, the sensitivity of 88.24 %, and specificity of 88.64 %. In another study, GLCM features are used to classify breast lesions on ultrasound (BUS) images (Gómez et al., 2012[51]) .

A particle swarm that optimised wavelet neural network (PSOWNN) is presented based on texture features for breast cancer detection in mammogram images (Dheeba et al., 2014[34]). Laws texture energy measures is employed to classify mass and calcification in digital mammograms. The achieved results show that the area under the ROC curve is 0.968 with a sensitivity of 94.1 % and specificity of 92.1 %. Dheeba and Singh (2015[33]) have developed a computer-aided diagnosis system known as Differential Evolution Optimized Wavelet Neural Network (DEOWNN) for automated breast cancer detection in mammography. The proposed system has used a series of texture features including GLCM feature, laws texture energy measures (LTEM) and Gabor features (GABOR). The obtained results show that the area under the ROC curve is 0.957 with the sensitivity of 93.3 %, specificity of 89.47 %, and accuracy 92.4 %.

### Classification

**Figure 7:**
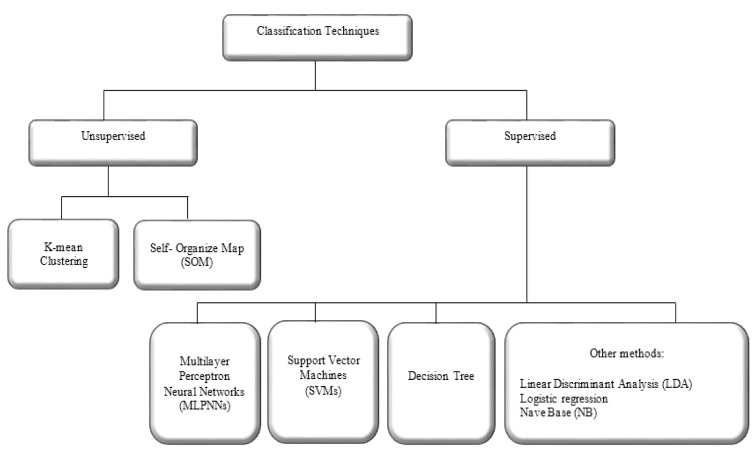
Overview of classification techniques

Classification is the last stage in CAD systems that differentiates and labels of the abnormality. Classification methods play an important role in the diagnosis and educational purposes in medicine. Classification approaches are categorised into two groups as shown in Figure 7(Fig. 7). Generally, in the implementation of classifier in clinical image processing, supervised classification techniques are used. 

Supervised classification examines a large number of unknown data and assigns them into related classes based on their characteristics. The main difference between unsupervised and supervised methods is that the unsupervised do not require pre-determined class. In a successfully supervised classification, all classes should be defined and the spectral properties of these classes have to be extracted during the training phase. However, in unsupervised classification, classes may be discovered but not known in advance. A brief description of the most popular supervised classification techniques along with their advantages and disadvantages are proposed in Table 3(Tab. 3). 

#### Impact of imbalanced data set in classification

The imbalance dataset is a crucial issue in various pattern recognition applications. In binary classification, this problem occurs when the number of instances from one class is significantly less than the other class. In this situation, the overall predictive accuracy is achieved by the majority class while the minority class has a greater impact on the classifier performance. The impact of the imbalanced data in the real-world applications is the irreversible effect on classification performance, specifically in medical diagnosis. Due to delays in diagnosis and treatment, the patient may lose their lives. 

To deal with the imbalanced dataset, several approaches have been presented in functional level and data level (Chawla et al., 2004[24]; Ganganwar, 2012[47]). Kernel transformation techniques and biased penalties approaches are recommended schemes for boosting support vector machines in functional level (Wang and Japkowicz, 2010[133]).

Kernel functions play a significant role in machine learning algorithms for effective linear or non-linear discrimination of high dimensional feature space. Available kernels for support vector machines include linear, polynomial, sigmoid and radial basis functions (Meyer, 2017[96]). Some studies have acquired that radial basis function (RBF) provide higher accuracy to other kernel functions particularly in nonlinearly separable training data (Abdi et al., 2012[3]; Anand et al., 2010[9]).

The cost-sensitive learning offers another way to address the imbalanced dataset issue. In cost-sensitive classification approaches, the sensibility of a classifier for a specific class can be increased by assigning a higher cost of misclassification to this class (Japkowicz and Stephen, 2002[70]). The misclassification cost can be manually offered by an expert or achieved by learning techniques (Sun et al., 2007[125], 2009[126]). A few cost sensitive learning schemes such as adaptive boosting cost sensitive, decision trees, and neural network cost sensitive have been introduced (He and Garcia, 2009[59]). The soft margin SVM is a biased penalised technique that provides an efficient solution to classify non-separable or imbalanced data (Pant et al., 2011[109]). Soft margin technique proposes a trade-off to minimise training error against margin boundary. In the case of non-separable data, soft margin classifier allows misclassifying some data points in the wrong side of the boundary.

At the data level, these solutions contain multitude variant forms of re-sampling such as random over-sampling, random under-sampling, directed over-sampling, directed under-sampling, and combinations of the above techniques. The rise of the likelihood for overfitting is the main drawback of random over-sampling techniques due to the replicating of minority instances (Almogahed and Kakadiaris, 2014[8]). Chawla et al. (2002[23]) proposed the Synthetic Minority Oversampling Technique (SMOTE) which is done by creating synthetic examples rather than by over-sampling with replacement. The minority class is over-sampled by taking each minority class sample and introducing synthetic examples along the line segments joining any/all of the k minority class nearest neighbours. SMOTE is an effective oversampling technique which has some deficiency such as over-generation because the generation of synthetic samples increases the classes overlapping (Almogahed and Kakadiaris, 2014[8]). Over-generation is problematic in the case of skewed class distribution with sparse minority class versus majority class (Maciejewski and Stefanowski, 2011[90]) . 

He et al. (2008[58]) present a novel adaptive synthetic (ADASYN) sampling approach for oversampling data to learn the imbalanced data sets. ADASYN is a weighted distribution method according to the level of difficulty in learning for different minority class samples. This sampling method measures the density distribution of minority instances to find a number of required samples to generate each minority instance. As a result, the ADASYN approach improves learning with respect to reducing the bias introduced by the imbalanced data distribution as well as autonomously shift the classifier decision boundary to be more centralised in those samples that are difficult to learn (Choi, 2010[26]; Haaland, 2013[53]). 

Random majority under-sampling is a common technique for under-sampling imbalance data (Seiffert et al., 2010[120]). The main drawback of the random under-sampling is the potential samples that may have been overlooked. One-sided selection is the effective way to improve the performance of random under-sampling. Kubat and Matwin (1997[79]) proposed a one-sided selection (OSS) in the attempts to intelligently under-sample the majority class by removing majority class examples that are considered either redundant or noisy. 

#### Classification methods in breast CAD systems

The selection of a reliable classifier is critical to succeed in distinguishing benign breast tumours from malignant ones. Different classification approaches have been developed for breast cancer detection in different modalities. Artificial intelligent techniques and support vector machines have been widely investigated to develop classification frame work in the diagnosis of breast cancer in recent years. A comparison of SVM, K-means cluster, and neural network have been presented in Liu et al. (2003[88]) to diagnose breast cancer. The outcomes indicate that the SVM exhibited a better whole performance. A computer-aided diagnosis system in ultrasound images has been developed in this study (Abdelwahed et al., 2015[1]). In this research the classification rate of SVM, K-nearest neighbour (KNN), and classification & regression tree (CART) have been compared. The results indicate that SVM and CART obtained higher classification rate rather than KNN in differentiating between normal and abnormal lesions. A comparative table of classification results obtained by other studies for breast cancer detection is presented in Table 4(Tab. 4) (References in Table 4: Chang et al., 2003[21]; Übeyli, 2007[132]; Bhardwaj and Tiwari, 2015[13]; Shi et al., 2010[122]; Dheeba et al., 2014[34]; Abdel-Zaher and Eldeib, 2016[2]). The performance of support vector machine (SVM) and multilayer perceptron neural network (MLPNN) to diagnosis breast tumour on ultrasound images has been presented in Chang et al. (2003[21]). The results show SVM provide higher accuracy, sensitivity and specificity in comparison to MLPNN in classification breast tumours. The breast cancer database from fine needle aspirates (FNA) from human breast tissue has been analysed in Bhardwaj and Tiwari (2015[13]) and Übeyli (2007[132]). The results illustrate that SVM present highest accuracy, sensitivity and specificity in comparison to combined neural network (CNN), probabilistic neural network (PNN) recurrent neural network (RNN) and MLPNN. The obtained results from Bhardwaj and Tiwari (2015[13]) indicate that genetically optimized neural network (GONN) provide highest accuracy, sensitivity and specificity in comparison with back propagation neural network (BPNN) and Koza's model. A Fuzzy Support Vector Machine (FSVM) proposed in Shi et al. (2010[122]) to detection and classification mass in breast ultrasound. The achieved outcomes are compared with decision tree (Horsch et al., 2002[61]) and LDA (Horsch et al., 2002[61]; Lefebvre et al., 2000[84]) methods. The outcomes show FSVM provide higher accuracy and specificity in contrast with decision tree and LDA. A Particle Swarm Optimized Wavelet Neural Network (PSOWNN) method proposed in Dheeba et al. (2014[34]) to classification mass on mammogram images. The performance measures compare with Swarm Optimized Neural Network (SONN) (Dheeba and Selvi, 2012[31]) and Differential Evolution Optimized Wavelet Neural Network (DEOWNN) (Dheeba and Selvi, 2012[32]) illustrated that enhanced accuracy, sensitivity and specificity has been achieved by PSOWN method.

### Performance evaluation

**Figure 8:**
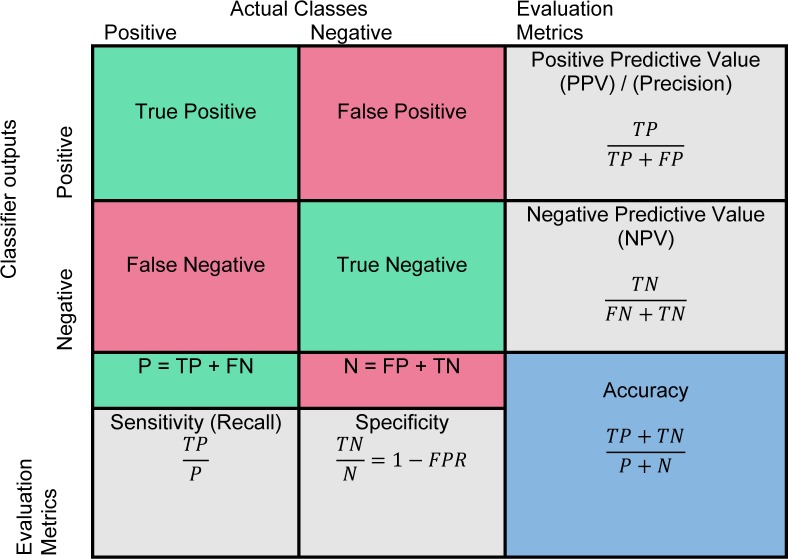
Confusion matrix and evaluation metrics

Evaluating the true performance of the classifier is the last stage in CAD system. The outcome of binary classification (Normal/Abnormal) is presented in a 2×2 confusion matrix in Figure 8(Fig. 8). True positive (Jinsamol et al., 2015[72]) value indicates the number of correctly predicted abnormality and true negative (TN) value shows the number of correctly predicted as a normal instance. The number of incorrect prediction of abnormality is shown by false negative (FN) and the number of incorrect prediction of normal objects is presented with false positive (FP) value. The performance of classifier has been generally measured by using various metrics (Fawcett, 2006[43]) such as sensitivity, specificity, positive predictive value (PPV), negative predictive value (NPV), and accuracy, in which the calculation is shown in Figure 8(Fig. 8). The main evaluation metrics that are used to assess performance of CAD systems include true positive fraction, false positive fraction, sensitivity, specificity, accuracy, receiver operating characteristics (Doi, 2014[37]; James et al., 2001[69]), and area under receiver operating characteristics (AUROC) (Gonçalves et al., 2014[52]). 

Receiver operative characteristics (James et al., 2001[69]) is a two-dimensional graph for visualisation, organisation, and selection of the classifier based on their performance (Fawcett, 2006[43]; Sonego et al., 2008[123]). The axes represent relative trade-offs between benefits (true positives) which are plotted on the Y and costs (false positives) which are plotted on the X (Fawcett, 2004[43]). Probabilistic classifier such as SVM and neural network return a score to depict the degree of belonging of an object to the specific class rather than other. These scores can be used to rank the test data and classifier to achieve the best performance if the positive samples are on the top of the list (Sonego et al., 2008[123]). The most advantageous ROC curve is compared to other metrics to assess the performance of a classifier is for the visualisation of classifier performance in all possible threshold. An ROC curve can be interpreted in two ways, graphically or numerically. A popular method to map an ROC curve to a single scalar value is the area under ROC curve (AUROC) (Bradley, 1997[17]; Hanley and McNeil, 1982[54]). 

## Conclusion

In recent years, CAD systems are developed to automate breast cancer detections and classification of benign and malignant lesions in different modalities such as ultrasound, mammography, and MRI. The CAD systems improve radiologists' performance in finding and discriminating between normal and abnormal tissues.

The main stages of implementation of CAD system and different techniques for each specific step were categorised and presented in this chapter. The region-based segmentation and clustering-based algorithms are wildly used to develop CAD systems for breast cancer detection. Extract suitable features for the detection of normal and abnormal lesions in breast depends on the nature of mass and imaging modalities, in which various features were introduced in this chapter. Artificial intelligent techniques and support vector machines have been widely investigated to develop classification frame-work in the diagnosis of breast cancer in recent years. 

The imbalance dataset is a crucial issue in various pattern recognition applications. To deal with the imbalanced dataset several approaches have been presented in functional level and data level (Ganganwar, 2012[47]). Kernel transformation techniques and biased penalties approaches are recommended schemes for boosting support vector machines in functional level. At the data level, over-sampling and under-sampling techniques are widely used to overcome imbalanced dataset issues. Synthetic Minority Oversampling Technique (SMOTE) and Adaptive Synthetic Sampling Approach (ADASYN) are effective oversampling techniques which have some deficiency such as over-generation because the generation of synthetic samples increases the classes overlapping. 

## Figures and Tables

**Table 1:**
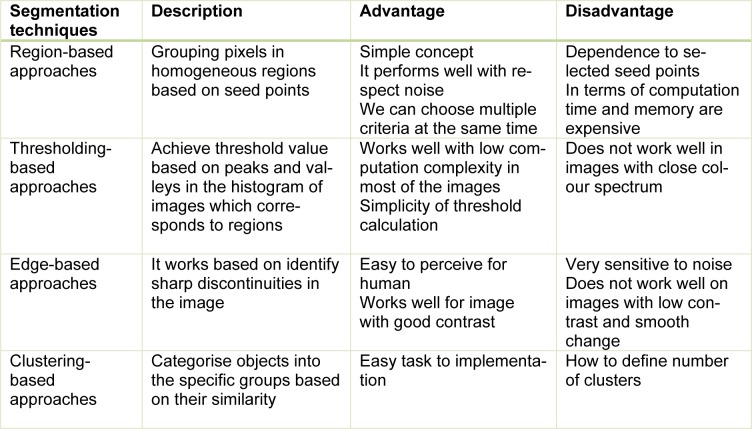
Comparison of segmentation techniques

**Table 2:**
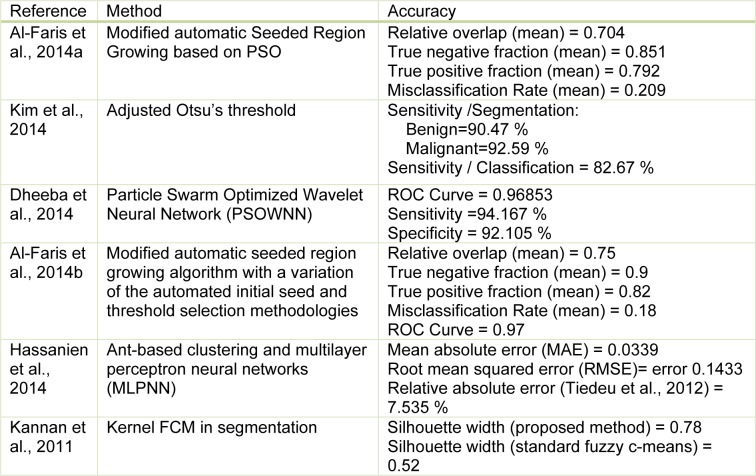
Segmentation techniques on recent studies in computer-aided diagnosis for breast cancer

**Table 3:**
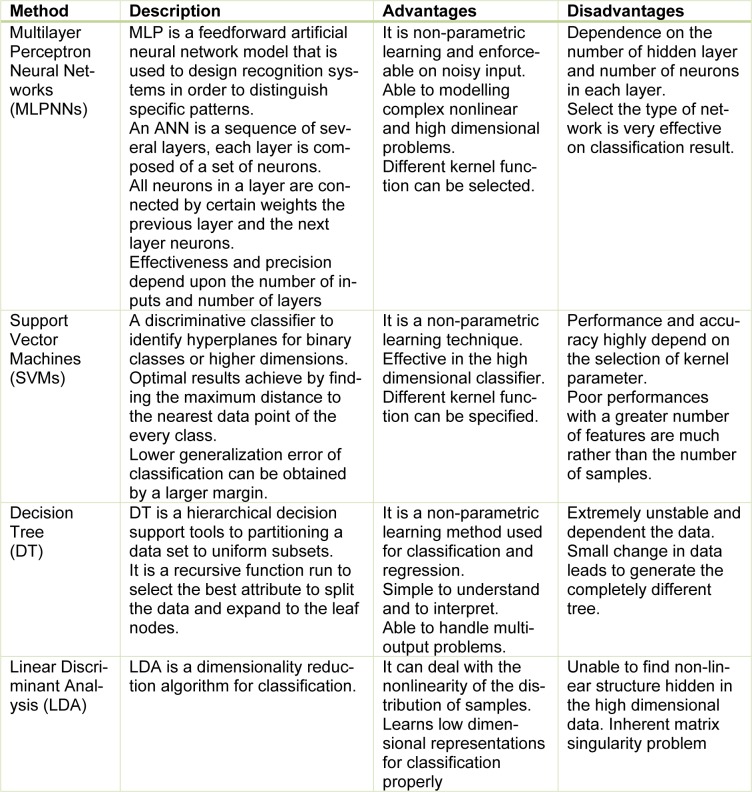
Description of supervised classification techniques

**Table 4:**
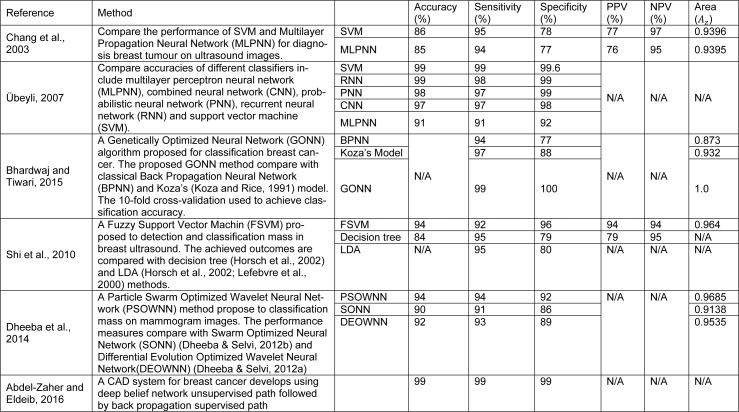
Classification techniques on recent studies in computer-aided diagnosis for breast cancer
